# Modeling Large Sparse Data for Feature Selection: Hospital Admission Predictions of the Dementia Patients Using Primary Care Electronic Health Records

**DOI:** 10.1109/JTEHM.2020.3040236

**Published:** 2020-11-24

**Authors:** Gavin Tsang, Shang-Ming Zhou, Xianghua Xie

**Affiliations:** 1 Department of Computer ScienceSwansea University 7759 SwanseaSA1 8ENU.K.; 2 Institute of Life Science, Swansea University 7759 SwanseaSA1 8ENU.K.

**Keywords:** Deep learning, dementia, electronic health records, feature selection, hospitalization, machine learning, risk factors, weight regularization

## Abstract

A growing elderly population suffering from incurable, chronic conditions such as dementia present a continual strain on medical services due to mental impairment paired with high comorbidity resulting in increased hospitalization risk. The identification of at risk individuals allows for preventative measures to alleviate said strain. Electronic health records provide opportunity for big data analysis to address such applications. Such data however, provides a challenging problem space for traditional statistics and machine learning due to high dimensionality and sparse data elements. This article proposes a novel machine learning methodology: entropy regularization with ensemble deep neural networks (ECNN), which simultaneously provides high predictive performance of hospitalization of patients with dementia whilst enabling an interpretable heuristic analysis of the model architecture, able to identify individual features of importance within a large feature domain space. Experimental results on health records containing 54,647 features were able to identify 10 event indicators within a patient timeline: a collection of diagnostic events, medication prescriptions and procedural events, the highest ranked being essential hypertension. The resulting subset was still able to provide a highly competitive hospitalization prediction (Accuracy: 0.759) as compared to the full feature domain (Accuracy: 0.755) or traditional feature selection techniques (Accuracy: 0.737), a significant reduction in feature size. The discovery and heuristic evidence of correlation provide evidence for further clinical study of said medical events as potential novel indicators. There also remains great potential for adaption of ECNN within other medical big data domains as a data mining tool for novel risk factor identification.

## I. Introduction

Dementia: a decline in mental ability severe enough to interfere with daily life. The primary cause of which being Alzheimer’s diseases making up 60–80% of cases [1]–[4]. Other causes include vascular dementia, thyroid problems and vitamin deficiencies [5]. Current estimates indicate 47.5 million individuals living with dementia in the world with predictions showing the figure to triple by 2050 [6], [7]. Around 100,000 individuals with dementia die each year [8], with a worldwide cost of 818 billion US Dollars in 2015 [9].

Dementia poses a significant increase in risk due to continued degradation of mental ability. As such, coupled with a generally higher level of comorbidity as compared to individuals with no dementia, it is often associated with adverse health outcomes resulting in higher rates of institutionalization and hospitalization [10]–[12], followed by lower survival rates [13]–[15]. Accordingly, the prediction of potential hospitalization of individuals with dementia allows for the identification of high-risk individuals in need of pre-emptive or preventative care.

With such a vast domain encompassed by the medical and social services potentially experienced by a patient, big data of such nature will invariably suffer from the *curse of dimensionality*, resulting in data domains consisting of upwards of thousands of dimensions. Consequent data sparsity follows behind as population size is vastly outpaced by the required sample size needed to maintain statistical significance for the size of feature space. For example, with over 100,000 potential medical event codes within the predominantly used ICD-10 system [16], healthcare data poses a significant challenge for the traditional statistical approaches generally applied within health informatics [17]. The use of such data within general predictive machine learning approaches poses additional challenges on interpretability and application on a human level. Without a reduction of feature size to a manageable size, the practicality of such approaches will remain outside of medical application, and firmly within the confines of academic interest.

To address such challenges, this article proposes a novel methodology for predicting hospital admission for individuals with dementia whilst simultaneously performing feature reduction on a sparse, high-dimensional dataset of medical events. The proposed methodology includes the use of a modified snapshot ensemble methodology originally proposed by [18] through the inclusion dynamic learning rate (LR) scheduling to produce a novel training methodology. The use of integrated entropy regularization [19], originally proposed for support vector machine (SVM), is also proposed with an adaption towards deep neural networks (NNs) used as the baseline modelling methodology within this study, henceforth referred to as ECNN.

By performing feature selection in parallel with classification training, selection of features can be focused on identifying effective discriminative features relevant primarily to the required task at hand. Being generic electronic health records of patient history without any direct relationship to dementia analysis or diagnosis, the reduction of the hundreds of thousands of potentially unrelated medical events to only a handful minimizes the number of redundant variables in need of further clinical or statistical study in identifying potential risk factors. The collection of electronic health records via the Secure anonymised information linkage (SAIL) data-bank [20] allows for the linkage of anonymized patient records across the various healthcare providers such as general practice (GP), in/out-patient hospital records, population deprivation, etc. This provides the potential of novel research applications involving the entirety of a patient time-line from birth to death.

## II. Related Work

Various studies have gone on to explore common causes of hospitalization within the population of dementia sufferers with a focus on clinical study and survey data with limited population scope. Kalisch *et al.* [21] identified, through a retrospective cohort study, a significantly increased risk of hospitalization for demented individuals when taking two or more anticholinergic medications with an adjusted incident rate ratio of 2.58. Chan *et al.* [22] follows a similar line of investigation indicating that 53.4% of cases of hospitalization of the elderly due to adverse drug events were preventable due to non-compliance or omission of indicated treatments. Phelan *et al.* [23] identified causes of hospitalization such as bacterial pneumonia, congestive heart failure, dehydration, duodenal ulcer and urinary tract infection as being significantly higher among those with dementia. Naalwala *et al.* [24] provides similar conclusions while also including causes such as bronchopneumonia. Bynum *et al.* [10] provides a more extensive list of hospitalization causes whilst also identifying the number of comorbidities as a consistent association with the odds of hospitalization. Toot *et al.* [25] establishes factors such as behavioral problems including agitation and wandering as well as changes in daily living routine to have an increased risk of hospitalization for people with dementia.

While the studies mentioned have provided informative results, the resulting causes of hospitalization all refer to a root cause in hindsight of the actual hospitalization event. Little research has been performed on identifying influential risk factors and clinical events from previous health records in an attempt to predict patient hospitalization. Related fields of research such as dementia diagnosis decision support systems have seen comparatively greater interest in the use of big data machine learning (ML) approaches. The resulting methodologies created from such fields of study provide great opportunity for adaptation into data mining and risk factor analysis.

Advances of information technology have led to a marked increase in information collected on patients in healthcare services. With surges in concepts of big data within other fields of research, ML applications are moving towards the forefront for data analysis. ML approaches within the field of medical informatics has already been ongoing with current research involving ML within dementia greatly focusing on early diagnosis with great success. The majority of the existing applications are based upon the analysis of magnetic resonance imaging (MRI) scan data which show impressive predictive performance. Marthotaarachchi *et al.* [26] uses a single PET & MRI scan to identify individuals with progressive mild cognitive impairment (MCI) as opposed to stable MCI. Through the use of a random forest (RF) and a novel random under-sampling methodology to resolve imbalanced class distributions, classification accuracy of 84% on a set of 273 patients was reported. Wolf *et al.* [27] tries to classify between individuals of MCI and full dementia based upon correlations between volume ratios of various brain regions. Brain regions were identified using boundary guided region growing whilst final classification was performed using a logistic regression model to reported accuracy of 78%. Lao. *et al.* [28] uses a novel mass preservation transformation methodology on MRI scans in addition to wavelet decomposition and SVM to classify between brain atrophy categories to produce a final accuracy of 87% over 153 patients. There exists several other studies involving MRI based dementia diagnosis [29]–[31]. The reliance on MRI scans for such methodologies, provides limited application in dementia diagnosis due to expense and availability of MRI technology [32]. As such, much akin to the established diagnosis procedure for dementia, cheaper more readily available methodologies such as Neuropsychological assessments remain the primary tool for initial mass screening.

Accordingly, Neuropsychological assessments such as the commonly used Mini Mental State Exam (MMSE) are used regularly as predictors for a ML based approach to patient screening [2], [33]. Maroco *et al.* [32] provides a thorough comparison of several ML methodologies including linear discriminant analysis (LDA), logistic regression (LogReg), SVM, RF and NN on a dataset of 10 neuropsychological tests with a sample size of 400 patients. With the task of classifying patients as having MCI or Dementia, results showed SVM with the largest overall classification accuracy. Meanwhile, more niche examples of ML within Dementia have shown highly remarkable results such as identifying semantic dementia patients through the use of natural language processing on descriptions of images made by demented and non-demented patients by Garrard *et al.* [34]. Using a naive Bayes multinomial algorithm, Garrard was able to classify dementia patients with an accuracy of greater than 90%.

Making use of limited datasets, the existing studies have provided encouraging results in dementia diagnosis. With further advances in information technology, the potential for large scale data analysis within medical informatics is apparent. Several major limiting challenges exist however; first and foremost being data protection and ensuring the privacy of an individual on a large scale. Secondly, with the timeline of an individual expanding across a range of discrete health and social service providers; the accurate, effective linkage of the various service database systems still presents as an ongoing research challenge [35], [36].

## III. Method

The proposed method, ECNN, consists of a four-stage pipeline: initial training using entropy weight regularization, snapshot ensemble training and aggregation, feature importance grouping and ranking, backward-stepwise feature selection & validation for risk factor analysis. The proceeding section presents initial data preprocessing following the individual pipeline stages in detail.

### A. Data Preprocessing

ECNN emphasizes the use of patient records consisting of GP read codes over a time period of multiple years. More detail of the experimental dataset is presented in section IV. All unique read codes were one-hot encoded as individual features with each patient sample indicating total occurrence of read code over the relevant time-period (see section IV). Data normalization of feature vectors to the range [0, 1] provides the final high-dimensional, sparse dataset for initial training. Class labels for samples are simply standard classification indicators, the set of {0, 1} indicating a positive or negative instance of any hospitalization event after official diagnosis of dementia as indicated within patient records.

### B. Deep Neural Networks

The foundational architecture of ECNN is the deep NN, commonly used in a wide selection of disciplines and research domains [37]–[42]. The remainder of this section provides a quick overview of NNs, highlighting the strengths over traditional statistical and machine learning methodologies already commonly in use within the health informatics domain.

Given an input space, $X \in \mathbb {R}^{n \times p}$ comprised of $n$ samples containing $p$ features: NNs consist of multiple layers of perceptrons, each aggregating the given input space through a weighted and biased sum before being mapped to an activation function. The result of which provides a final activation output shown in the following equation:

\begin{align*} a^{l=1}_{k}=&\sigma \left ({\sum _{j=1}^{p}w^{l}_{j} a^{l-1}_{j} + b^{l}_{j}}\right), \\ a^{0}_{j}=&X_{j}\tag{1}\end{align*}

where weight, $w$ and bias, $b$ are trainable parameters transforming each input feature vector, $X_{j}$ before summation and transformation using activation function, $\sigma $ to provide the indexed perceptron $k$’s, activation result $a$ within the first layer, $l$. The vector of activations, $a^{l}$ are passed as the input vector for the subsequent layer of perceptrons. A deep NN constitutes an architecture containing multiple preliminary embedding layers between the input and output layer allowing for non-linear transformations and subsequent embeddings of the original data space. Each snapshot NN within the overall ECNN architecture consists of a 2 hidden layer architecture containing 50 and 30 perceptrons accordingly. Perceptron counts were chosen using a simplistic grid search hyper-parameter optimization algorithm to provide best model performance.

Training consists of the minimization of the cross entropy cost function:

\begin{equation*} \min _{\hat {y}} [-(y \log (\hat {y}) + (1-y) \log (1-\hat {y})) + \lambda f(w)]\tag{2}\end{equation*}

based on the forward-pass and back-propagation methodology to adjust model parameters in (1), regularized by the $\lambda $ weighted function $f(w)$; where $\hat {y}$ is the model probability output and $y$, the classification target. Said regularization will be the aforementioned entropy weight regularization function examined in Section III-C.

### C. Entropy Weight Regularization

As mentioned previously, dimensionality and sparsity are the main challenges of data analytics using electronic health records. With data dimensionality potentially numbering in the hundreds of thousands and individual observations having perhaps tens of values, the leveraging of such data in producing an effective predictive model whilst maintaining comprehensibility is a hard prospect.

Traditional dimensionality reduction pipelines such as principal component analysis (PCA), relying on orthogonal transformations of the dataset, suffers on a comprehensibility standpoint. After said orthogonal transformation into the new embedding space, with axes not necessarily parallel to the original feature space axes and based off orthogonal vectors of most variance, each of the resulting orthogonal dimensions or principal components become fully dependent on every original feature.

After the removal of low-variance principal components, the traditional methodology for PCA dimensionality reduction, a transformation back into original feature-space would result in a information loss across multiple features due to the aforementioned dependence. Consequently, the selection of a single principal component of high-importance would transform into a vector spanning across the entire feature space. Subsequent selection or ranking of individual read codes for clinical significance would thus become highly impractical.

Furthermore, a final application involving the use of such dimensionality reduction methodologies will still require the evaluation of every medical event within a patient time-line. Another major disadvantage of such methods is the apparent disconnect between dimensionality reduction and prediction. PCA bases dimensionality reduction on the variance of a dataset and as such performs reduction without any feedback as to its effectiveness.

The method proposed below seeks to solve both issues. By performing feature selection during the training of the predictive model, feedback on the performance of the predictive model based upon the reduced features can be fed back into selecting features relevant to the trained task at hand. In addition, reduction will be performed directly on feature dimensions and as such, allows for the direct removal of redundant events within a patient time-line.

This article proposes a novel adaption of the entropy regularization technique, originally proposed by Zhou *et al.* (3) for SVM models, towards the NN architecture. The measure of information entropy defines the potential information content of a data source or the unpredictability of a certain state occurring. As such, within a probability mass function, $P(X)$, of a binary variable, $X$, the information entropy of said variable will approach zero where the probability mass function approaches near certainty of one or the other action. The information entropy is highest at the midpoint, $P(X)=0.5$, where the probability of either action is exactly equal. Consequently, this property of information entropy can be leveraged into enforcing weight sparsity within our methodology.

By incorporating entropy regularization based on the bounded weights of the first layer of the NN within the cost function, weight updates will seek to minimize entropy, thus driving said first layer weights towards {0,1}. The original cost function seeks to push weights in either direction towards improving predictive accuracy. With a linear activation function, weights approaching zero will filter out activation signals whilst weights approaching one will remain unaltered. Entropy regularization will emphasize the need to push weights towards boundary extremes. The combination of the aforementioned functions will result in activation signals of importance being driven towards one whilst redundant signals in the scope of predictive performance will be pushed towards zero and thus filtered out. The resulting weight matrix will be of a sparse form consisting of only activation signals which contribute to the model prediction.

\begin{equation*} f(w) = -\lambda \sum _{jk}^{JK}w_{jk} \log (w_{jk})\tag{3}\end{equation*}

where $W_{jk}^{1}$ is the weight representing the connected edge between the $k$-th multilayer perceptron (MLP) in layer $l$ and the $j$-th MLP in layer $l-1$. The hyper-parameter, $\lambda $ is a regularization coefficient to fine-tune the balance between predictive performance and weight sparsity. Consequently, weights close to zero will map to $\theta =0$ while highly positive weights will map towards $\theta =1$. The resulting sparse weight matrix of the first layer will act as a filter, removing inconsequential connections between MLPs within the first and second layer. By evaluating this matrix, the resulting input features can be categorized into three types shown in ascending order of importance:

In reference to the traditional cross entropy cost function for overall prediction cost minimization:

\begin{equation*} C=-(y \log (\hat {y}) + (1-y) \log (1-\hat {y})) + \lambda _{0} f(w)\tag{4}\end{equation*}

where model prediction, $\hat {y}$ are driven towards

#### 1) Disconnected

Features whose weighted connections have been driven close to zero are completely excluded from the remaining model and as such, are non-meaningful features for classification.

#### 2) Partially Connected

Features where only some weighted connections have been driven close to zero. Consequently, these features exhibit element-wise sparsity and as such remain partially used.

#### 3) Fully Connected

Features whose weights exhibit non-sparsity indicates a favorable feature which remains in use for the remainder of the model.

By selecting favorable features whose associated weights are large whist being fully or partially connected allows for redundant features to be removed. Through associating feature selection based upon parameters within the predictive model during training, feature selection can be tailored towards selecting features which favor heavily into the overall predictive performance.

### D. Snapshot Ensembles

The training procedure used involved the use of a modified snapshot ensemble training procedure proposed by Huang *et al.* [18] allowing for multiple ensemble NNs to be generated through training a single model. Ensembles comprise of periodic model *snapshots* taken during training. Diversity between each model snapshot is encouraged through specific LR scheduling between each snapshot. Specifically, a cyclic cosine function [43] repeating based on set training iterations:

\begin{equation*} \alpha (t)=\frac {\alpha _{0}}{2}\left ({\cos \left ({\frac {\pi ~\text {mod}\left ({t-1,\lceil \frac {T}{M}\rceil }\right)}{\lceil \frac {T}{M}\rceil }}\right)+1}\right)\tag{5}\end{equation*}

where the LR, $\alpha $, is dictated by scaling the original LR, $\alpha _{0}$, based off the current epoch, $t$’s position within the shifted sub-cosine function. Each of the $M$ number of cosine functions is spread equally along to the total epoch count, $T$.

The resulting LR progression over a cosine cycle resembles a rapidly descending LR from an initial large value, gradually reducing in gradient to a set iteration and an assumed model convergence at local minima. At which point, model parameters are saved as a single ensemble snapshot before a large spike in LR is introduced to repeat the cosine cycle. Said LR spike “dislodges” the model from the local minima allowing for descent into a potentially new local minima and resulting new unique ensemble model.

The resulting unique snapshot sub-models form a large combined final model for use in the testing stage. Final predictions are formed from the combined predictions of each snapshot model based off the combined average.

The result of which, as indicated by Huang *et al.*, provides superior model accuracy and generalizability with similar training durations as compared to traditional momentum based learning rate schedulers. Such behaviour additionally provides potential to encourage divergent sparse first layer weights in combination with the aforementioned entropy weight regularization (See Fig. 6). The result of which, provides diverse feature combinations for analysis.

**FIGURE 1.**
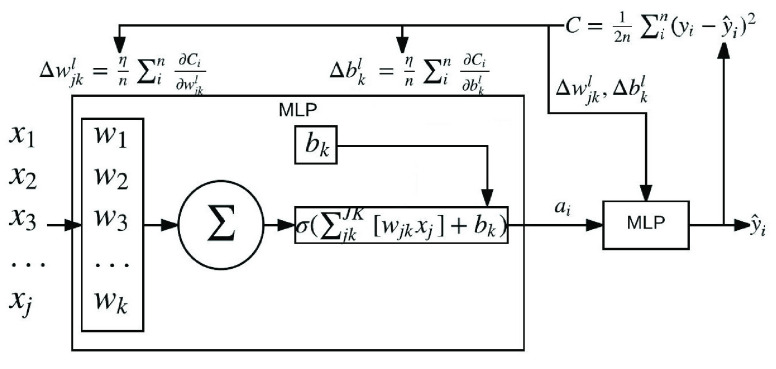
Exploded view of a perceptron contained within a NN architecture consisting of an input, hidden, and output layer. Also shown, are the forward pass formulae for producing overall model loss using an example mean squared error loss function. Additionally, back-propagation formulae are also shown, used for updating network parameters, perceptron weight, $s$ and bias $b$. To note, is the propagation of remaining error being passed back up each perceptron layer via partial derivatives, $\frac {\delta C_{i}}{\delta b^{l}_{k}}$ and $\frac {\delta C_{i}}{\delta w^{l}_{jk}}$ for weight and bias respectively.

**FIGURE 2.**
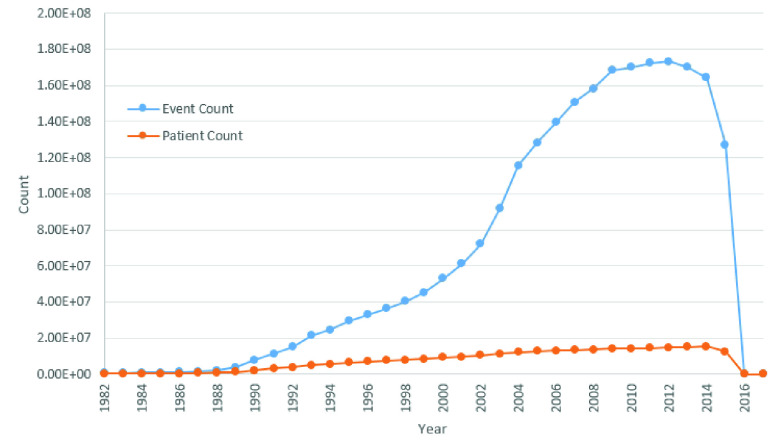
Graph indicating distribution of patient and event counts aggregated by year across the GP and PEDW datasets used for evaluation. As shown, the majority of patients and events span across a timeframe between 1982 to 2015. Of note, is the non-linear correlation between patient count and event count highlighting an increased frequency of recorded events over the years.

**FIGURE 3.**
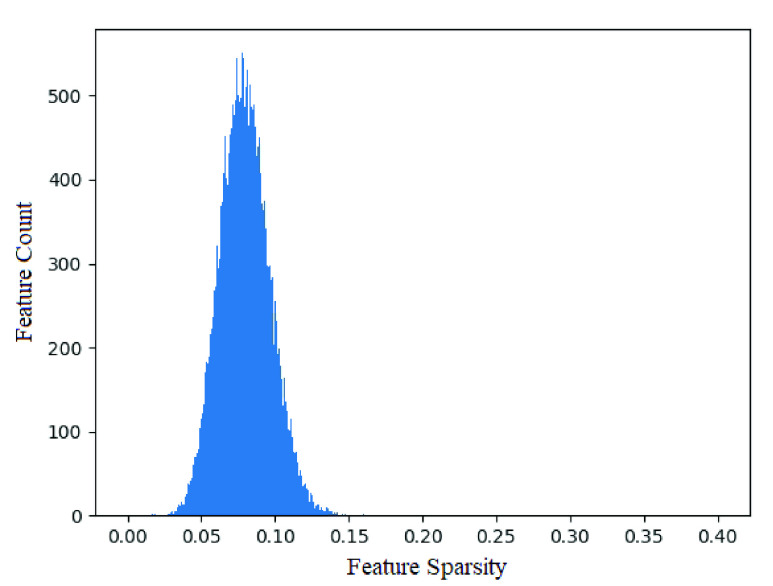
Histogram of features over mean importance factor across all snapshot ensembles of a randomly selected cross-validation run. As seen, the majority of features are normally distributed ($\mu =0.0777, \sigma =0.0265$) around a low overall feature sparsity value, indicating the majority of features introduced to ECNN are of low importance in prediction of hospitalization. Unable to be effectively shown, due to graph scaling constraints, 10 features lie outside 3 standard deviations of the distribution, shown in table 4.

**FIGURE 4.**
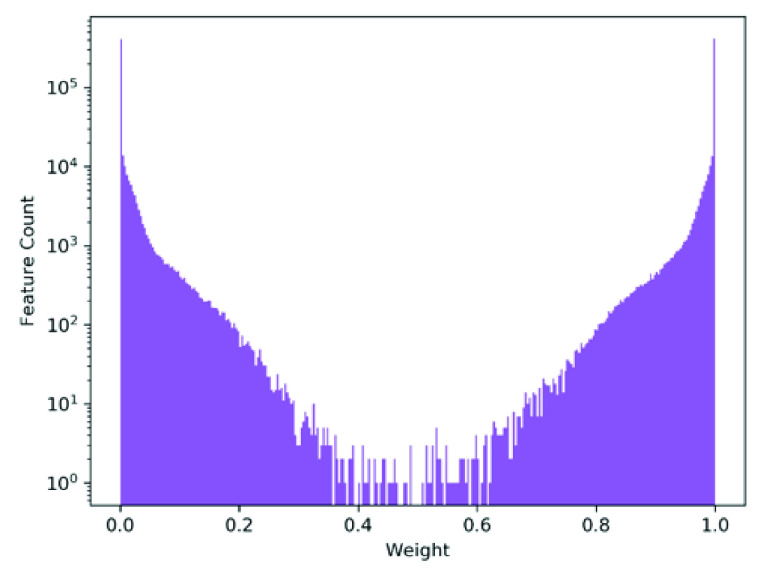
Shown, is the log scaled histogram of final model weights of the first layer of a randomly selected model within cross-validation. As seen, the vast majority of weights have converged to values close to {0, 1} in response to the proposed entropy weight regularization. As mentioned, a comparatively small set of weights (an order of magnitude less than successfully separated) show a non-perfect separation towards either extreme. Further analysis of said weights indicate belonging to specific features, contributing to the ultimate variance between each ensemble, as highlighted in Fig. 6.

**FIGURE 5.**
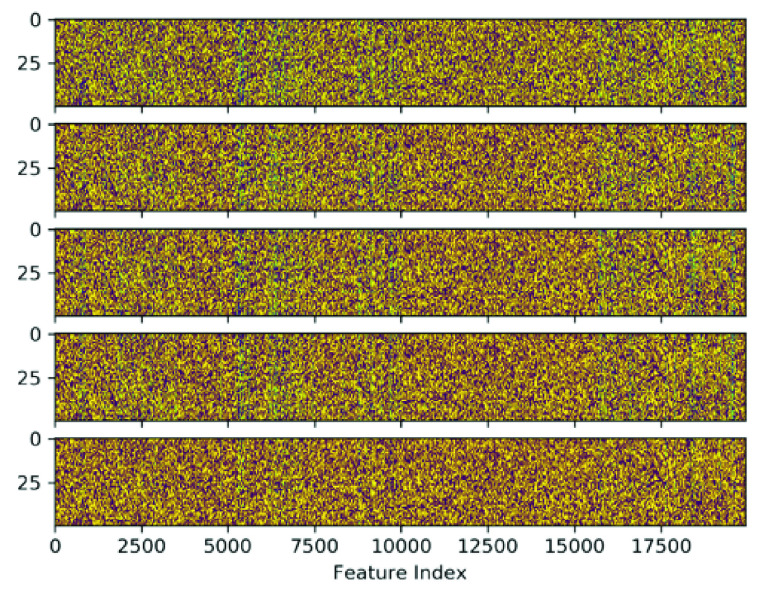
Heat map of individual weights of the first layer across the five snapshot ensemble models of a single cross-validation run where yellow hot are values close to 1 and purple cold are values close to 0. As seen, most weights have converged to values close to {0, 1} indicating the success of entropy regularization. Rough patterning between each heat map indicates a potential pattern in variation between each ensemble. As similarities and differences between each ensemble weight matrix are difficult to see, Fig. 5 provides a clear indication of the feature-wise variation.

**FIGURE 6.**
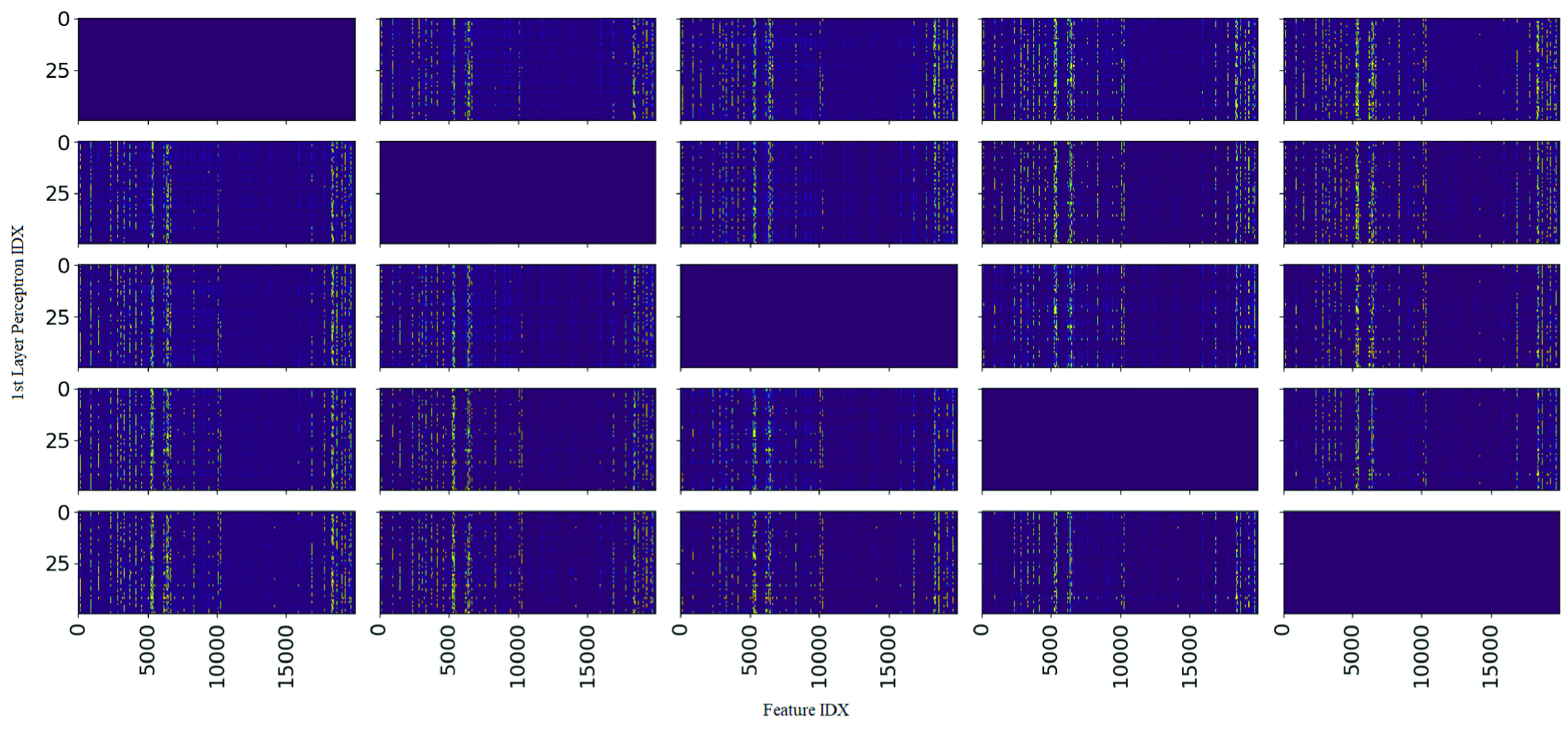
Shown, is a complete comparative heat map matrix of the absolute differences in weights between the first hidden layer of every possible pair of the 5 produced snapshot ensembles. Colour values are mapped so that yellow hot represents values close to 1 whilst purple cold represent values close to 0. Left-top to right-bottom diagonals show weight difference between the same ensemble and are thus irrelevant for analysis. Of note, the vertical patterning for each heat map indicates any weight differences between snapshot pairs are focused on specific individual features across all snapshot pairs. This suggests a convergence in importance factor for the vast majority of features with a small but consistent subset of edge-case features producing variation amongst the snapshot ensembles.

### E. Feature Ranking & Selection

Features can be categorized based upon the sparse weight matrix into three categories as detailed in section III-C. An evaluation metric was designed as shown in (6) called *Feature Sparsity Importance* to provide the capability to rank and identify possible features. Overall, feature ranking is based off the perceptron weight parameters directly associated to each feature between the input and first hidden layer of each snapshot using the following equation:

\begin{equation*} R_{k}=\frac {\overline {|W_{k}^{1}|} - \sigma ^{2}(|W_{k}^{1}|)}{\max (\overline {|W_{k}^{1}|})}\tag{6}\end{equation*}

where $\overline {|W_{k}^{1}|}$ is the mean absolute weight on a column by column basis representing the mean weight associated with feature $k$. A higher mean absolute weight will generally indicate a feature of higher importance. In order to account for element-wise sparsity within the weight matrix, the variance of the absolute weights, $\sigma ^{2}(|W_{k}^{1}|)$, is also taken into account:

\begin{equation*} \sigma ^{2}(W^{l}_{k})=\frac {\sum _{j}^{J}(w^{l}_{jk}-\overline {w}^{l}_{k})^{2}}{J-1}\tag{7}\end{equation*}

where a high value indicates high element-wise sparsity and vice versa. The maximum mean absolute weight used within the denominator ensures a non-dimensional value normalised to 0, 1. The feature sparsity importance metric will evaluate fully connected features with high mean and low variance highly, partially connected features with high mean and high variance lower and finally disconnected features of low mean and low variance to a value near zero indicating low overall importance to the predictive model. Feature importance values from each snapshot model were averaged to obtain the final Feature Sparsity Importance value for use in thresholding.

Feature thresholding can be performed using various schema. Methodologies such as selecting based off a 95% importance cut-off would provide an effective adaptive threshold emphasising predictive performance. Such a cut-off would however produce a 107 feature subset, whilst a significant reduction, would still remain cumbersome in an application standpoint. A simple top $k=10$ cutoff threshold provides a rather naive threshold policy, however coincidentally, as shown in Fig. 3, a normal distribution fitted across a feature importance histogram highlights the predominance of low importance features whilst 10 features lie high outside the three standard deviation range. As such, these features are selected as the subset for further analysis.

Such feature ranking within the original data space contrasts highly with traditional statistical modelling techniques such as PCA or LDA requiring orthogonal transformation into an embedding space for dimension ranking. As such, ECNN enables a direct interpretable ranking of individual medical events as predictive indicators of future hospitalization.

## IV. Experiment

The dataset population was extracted through the SAIL data-bank which consists of linked and coded patient records catalogued from various primary and secondary health services provided by the Welsh NHS, UK. Accordingly, data coverage encompasses the majority of the Welsh population, a total of 3 million individuals [44].

The Primary Care GP dataset (GP) contains individual medical records obtained from the various primary care practices around Wales. Every individual contains timestamped records of various events ranging from prescribed medication to lab test results to diagnoses coded as NHS read codes. The Patient Episode Database for Wales (PEDW) dataset comprises of attendance and clinical information for all hospital admissions within Wales. A continuous period of treatment for an individual can be traced from entry to diagnosis to hospital transfer, if any, to treatment to exit. Information such as date of birth, gender, area of residence, deprivation score, etc. are provided if available for both datasets.

Data preparation involved the selection of all patients with a positive diagnosis of dementia based upon NHS read codes as indicated in Table 1 [45]. Of note is the hierarchical nature of said read codes allows for a general broad consolidation of dementia diagnosis for simplification. Such examples include codes such as ‘E00..’ indicating all variations of code values possible on positions containing the decimal point. In practice however, there is inconsistent inclusion of both categorical and sub-categorical read codes within the dataset. As such, all categorical and sub-categorical read codes for dementia were included to ensure thorough consideration of all indicated dementia patients.

**TABLE 1:** Table Containing Read Codes Associated With a Positive Dementia Diagnosis

Read Codes					
E00..	E003.	Eu001	Eu021	E000.	E004.
Eu002	Eu022	E001.	E0040	Eu00z	Eu023
E0010	E0041	Eu01.	Eu024	E0011	E0042
Eu010	F11x2	E0012	E0043	Eu011	F11x5
E0013	E004z	Eu012	F11x6	E001z	E012.
Eu013	F11x7	E002.	E0120	Eu01y	F11x8
E0020	E041.	Eu01z	F11x9	E0021	Eu00.
Eu02.	F11xz	E002z	Eu000	Eu020	Fyu30

The overall dataset consists of the medical history from 1908 to 2017. However, dataset distribution by year as shown in Fig. 2, indicates the vast majority of patient events distributed between 1982 to 2015. As such, patients and corresponding records have been limited to the aforementioned time window. The selected population variation results in a gender split of 34.9% male and an overall mean age of 91.8 and a standard deviation of 10.50. The generally older population characteristic of our dataset provides opportunity for analysis into an especially more vulnerable age range of the general population more prevalent to dementia and resulting hospitalization or institutionalization.

Further statistical population characteristics are shown in table 2.

**TABLE 2:** Statistical Characteristics of Sampled Population

Category	Gender	Cond. +ve	Cond. −ve	Total
Mean Age	Male	85.18±9.44	93.62±10.56	89.04±10.82
	Female	88.98±8.51	97.41±9.62	93.27±10.02
	Total	87.56±9.05	96.19±10.09	91.80±10.50
Mean Event Count / Person	Male	1256±1127	443±565	885±1000
	Female	1318±1220	469±624	886±1053
	Total	1295±1187	461±605	872±884
Population	Male	11233	9441	20674
	Female	18945	19679	38624
	Total	30178	29120	59298

The resulting dataset consists of 59,298 patients diagnosed with dementia. Patient time-lines were selected one year before dementia diagnosis up to hospital admission if at all. An individual patient history, or sample within the input dataset consists of a frequency table counting number of times specific medical events occur in a one year lead up to first hospitalization event. With a formatting similar to traditional one-hot encoding, the feature set comprises of all possible unique medical events which have occurred within the considered population resulting in 54,649 unique features or event codes. Whilst, the sum total frequency of all occurring medical events with in the population totals 52.5 million events, with a single individual medical history only using a small subset of said unique events, a significantly sparse dataset is produced, effectively highlighting the challenging extent of high dimensionality and data sparsity inherent within patient medical histories constructed into datasets for ML modelling. Consequently, such dataset properties provide an excellent opportunity for verification of ECNN.

As mentioned previously, the evaluation criteria for our methodology will be in predicting whether a dementia patient stays within a GP setting with minor accidents and events (condition negative) or whether a patient is admitted into a hospital setting due to major accidents or continued degradation of mental ability (condition positive). This will be indicated through a lack of hospital data throughout a patient’s time-line. The resulting patient dataset split consists of 30,178 patients admitted to hospital and 29,120 patients which remained within a GP setting.

A comparative evaluation between a similar traditional classification model with capability for feature ranking, RF was performed using the exact same dataset. Feature ranking on RF was produced through the use of traditional out-of-bag error comparison to perturbed datasets [46]. Additional comparative evaluation was also performed with a baseline methodology through a subset of 10 random features selected amongst the original overall feature-set via random number generator.

## V. Results

Experimental evaluation can be categorized into three distinct categories: predictive performance using the full dataset (section V-A), analysis of model characteristics to produce a feature ranking (section V-B), and final evaluation of feature ranking and selection against baseline methods (section V-C. All experimentation was cross-validated using a 5 fold, traditional k-fold validation paradigm. In which, three folds are designated as the training set, one for validation and one for final testing in a cyclic sequence; repeated twice over. The resulting $5\times 2$ test fold sequences of results are aggregated and presented within the remainder of this section.

### A. Full Feature Results

The performance of ECNN as a pure classification model was assessed on the full set of features in comparison to a traditional classification methodology with combined feature ranking capability, RF. The intuition of such an assessment, in combination with section V-C, being the evaluation of the validity of resulting feature rankings from ECNN.

Results are presented in table 3 showing aggregated predictive performance across various metrics with T-test to distinguish significance between the two methodologies. As shown, ECNN provides significant improvements (<0.05 P-value), around 5%, in true negative rate (TNR) and positive predictive value (PPV) compared to RF whilst maintaining insignificantly near similar performance in true positive rate (TPR) and negative predictive value (NPV) resulting in an overall superior model performance in accuracy. A major consideration however, is the larger variation in predictive performance of ECNN as compared to RF. Such variation was found during testing to be caused in part from the use of entropy regularization settling into perhaps a sub-par local minima of sparse weights producing inferior performing model snapshots affecting overall stability during the final prediction aggregation of the ensemble models.

**TABLE 3:** Full Feature Set Classification Results

Evaluation Metric	ECNN			RF			P-Value
	Mean±Std. Dev.	95% CI		Mean±Std. Dev.	95% CI		
TPR	0.746±0.036	0.719	0.773	0.746±0.005	0.742	0.750	0.986
TNR	0.762±0.043	0.729	0.794	0.714±0.007	0.709	0.718	0.004
PPV	0.766±0.024	0.748	0.785	0.710±0.005	0.706	0.713	2.27E-06
NPV	0.744±0.019	0.730	0.758	0.750±0.003	0.747	0.752	0.404
Accuracy	0.755±0.005	0.750	0.757	0.729±0.002	0.728	0.731	2.61E-11

Comparative analysis of predictive performance between ECNN and random forest - a traditional classification model with the capability to perform feature ranking and selection. As shown, ECNN provides statistically significant, superior accuracy whilst providing superior feature selection (see table VII)

The resulting overall performance improvement over RF however, comes with a major compromise of training complexity and duration as is standard in a comparison of RF to NN trade-offs. With a significant difference between RF and ECNN of 44 seconds to 2 hours average training duration respectively, such vast differences highlights the greatest disadvantage of ECNN and deep NN complexity overall. However, with a significant improvement in both predictive performance and feature ranking capability, as shown in section V-C, such performance may justify the differences in training times.

### B. Feature Selection

Within this section, we will present and analyse the resulting ensemble snapshots using the aforementioned feature ranking metric presented in section III-E.

As shown in Fig. 3, entropy regularization was able to successfully separate the majority of layer weights into a sparse filter mapping of values close to zero and one. Fig. 5 alternatively provides a heatmap representation of the sparse first layer weights of each snapshot ensemble model produced. As seen, each ensemble mostly resembles each other with subtle differences highlighted in Fig. 6 showing normalized difference of first layer weights between each pair of ensemble models. As such, snapshot ensembles are shown to successfully dislodge settled weights to generate new feature maps. Of note is how weight variance between ensembles centers around specific features; as opposed to across layer 2 nodes or a combination of both. Consequently, such behaviour can be interpreted as high feature variance between ensembles indicating uncertainty of feature importance whilst low variance indicates a convergence of such features into a stable configuration of importance.

The proposed feature ranking metric was applied to the first layer weights of each ensemble and aggregated into a single normalized feature importance value for each individual feature. Fig. 3 indicates the distribution of features across the feature importance spectrum. As seen, the majority of features form a normal distribution low on the feature importance metric with mean, $\mu =0.0777$, and standard deviation, $\sigma =0.0265$; whilst several features lie high on feature importance outwith the normal distribution by greater than three standard deviations. Consequently, these 10 outlier features were selected as the subset of important features used for continued further analysis, in addition to subset predictive performance testing in section V-C.

These 10 medical events, summarized in table 4, form a varied collection of medical diagnoses, medication prescriptions and procedural events. Qualitative analysis and literature review of the identified medical events show effective feature selection from ECNN with every event occurrence being either positively associated to an increased hospitalization risk or present an entirely novel or inconclusive association.

**TABLE 4:** Top 10 Event Codes Ranked in Order of Importance as Determined by ECNN

Importance	Event CD	Definition	Pearson’s $r$	P-Value
0.481	G20..	Essential Hypertension	0.1504	4.65E-297
0.318	ip3j.	Adcal-D3 1.5g/10ug chewable tablet	0.0695	1.98E-64
0.300	n473.	Influvac sub-unit prefilled syringe 0.5mL	0.0635	4.44E-54
0.259	44Uz.	Blood glucose raised NOS	0.0223	5.71E-08
0.254	bxd5.	Simvastatin 40mg tablet	0.1879	0.0
0.247	1323.	Social group 3 - skilled	0.0040	0.326
0.234	dh12.	Serc-16 Tablet	0.0107	9.39E-03
0.234	ja1I.	Ibugel gel 100g	0.0154	1.71E-04
0.231	E2749	Nightmares	0.0070	0.087
0.227	9N32.	Third Party Encounter	0.0626	1.23E-52

A Pearson’s $r$ statistic was used to measure linear correlation between medical event occurrence and hospitalization incidence in dementia patients. As shown, most of the ranked events show statistically significant linear positive correlation to hospitalization incidence highlighting potential predictors of hospitalization events post indication of such medical events. Of, note is the ranking of event codes based off $r$ statistic being non-similar to the ranking produced by ECNN. Of course, with $r$ statistic analysing only linear bivariate correlations, any non-linear multi-variate associations between event and hospitalization incidence detected by ECNN are lost, explaining the discrepancy between $r$ statistic ranking and ECNN ranking. Multi-variate statistical analysis is further warranted on the identified events is a potential avenue of further investigation.

In regards to established direct risk factors identified by ECNN, a literature review is presented highlighting each positive correlation. As shown, a diagnosis of essential hypertension or idiopathic hypertension was identified as the highest ranked feature with an average importance factor of 0.481, vastly exceeding the exhibited normal feature distribution mentioned previously. Of course, such a correlation between hypertension and hospitalization incidence has already been shown to exist through cohort studies [47], [48]. Previous literature have also studied several other risk factors identified by ECNN. In regards to the second most highly ranked event, prescription of *Adcal-D3 - calcium and vitamin D* supplements, under the assumption of a resulting vitamin D or calcium deficiency in the individual, studies have shown general increase in hospitalization risk for the elderly from resulting co-morbidities [49] in addition to direct potential risk [50], [51]. *Influvac*, a flu vaccine, the third highest ranked event, regularly prescribed to highly at risk elderly individuals, highlights established risk factors of influenza on functional decline within the elderly [52]. Additionally, blood glucose lab tests for potential diabetes and *simvastatin*, prescribed for high blood cholesterol are further established risk factors for general hospitalization risk in the elderly demented population [48]. Osteoarthritis, a condition with a common prescription of *Ibugel* [53] - a gel based ibuprofen medication identified as 7th on the list, is also widely regarded as a hospitalization risk factor of the elderly [54].

Prescription of *Serc-16 tablets*, prescribed for Ménière’s disease, presents an interesting secondary indicator of hospitalization risk. With symptoms of vertigo, titinnus, and hearing loss - Ménière’s disease associates with increased fall risk in the elderly [55] resulting in indirect risk of hospitalization.

As shown, the identification of already established risk factors by ECNN demonstrates effective risk factor recognition, highlighting the potential for further clinical analysis on the remaining medical events for potential correlations. Of the remaining event indicators: *Social group 3 - skilled*, occurrence of nightmares and encounter between GP and a third party in regards to the patient; little or inconclusive studies have attributed such events as a precursor to hospitalization. Van de Vorst *et al.* indicates no statistical significance for hospitalization risk between mid-tier socioeconomic status, generally associated with a skilled individual, and high or low-tier status. There was however, positive significant correlation from low to high-tier status [56]. Nightmares have potential to be associated with symptoms of delirium, the result of which, hospitalization risk is increased [57]; however, such a generic medical event with multiple associations to various conditions would require further study to be presented as an indicator on it’s own. Finally, third party encounter addresses a wide range of situations involving reports by individuals related to the individual suffering from dementia. Whilst it has been established that dementia detection is predominantly reliant on self-reporting or by relatives [58], no literature was found studying hospitalization resulting from non-emergency third party reports.

Linear independent correlations between the identified medical events to hospitalization incidence was analysed through Pearson’s correlation and reported in table 4. Interestingly, there seems to be little correspondence between $r$ value and ECNN ranking and in some cases, little statistical significance. Such behaviours indicate a distinct lack of independent linear correlations on individual risk factors. Tests on modelling hospitalization prediction using NN and RF on the individual, identified features provide no discriminative capability; requiring all 10 features to produce predictive performance indicated in section V-C. Such observation hints at the capability of the underlying NN architecture of ECNN being able to formulate non-linear relationships between features, consequently being unable to produce individually discriminative medical events. The extraction and interpretation of non-linear combinatorial relationships between features remains an open avenue for further research of great benefit within the medical informatics field.

### C. Reduced Feature-Set Predictive Performance

Several comparisons were evaluated to determine feature selection performance. The reduced subset of features produced by ECNN were used to train on various standard classification methodologies as a comparison to the full dataset. The top 10 features ranked by RF, shown in table 5, were also used as a baseline comparison of a traditional effective feature selection procedure while a random 10 feature selection was also evaluated to provide an indicator of dataset baseline predictability. The results are shown in table 7.

**TABLE 5:** Top 10 Event Codes Ranked in Order of Importance as Determined by Random Forest

Importance	Event CD	Definition
0.0164	44M3.	Serum total protein
0.0162	42L..	Basophil count
0.0136	44D6.	Liver function test
0.0112	44M4.	Serum albumin
0.0100	451E.	GFR calculated abbreviated MDRD
0.0085	44P5.	Serum HDL cholesterol level
0.0083	44I5.	Serum sodium
0.0083	44I8.	Serum calcium
0.0080	1Z12.	Chronic kidney disease stage 3
0.0071	9N36.	Letter from specialist

Whilst RF highlighted entirely separate features of importance compared to ECNN, several event codes can be hypothesised to indicate similar clinical significance. For instance, serum calcium indicates the use of calcium level blood tests potentially related to the dispensing of Adcal-D3 tablets as indicated by ECNN. Similar indications can be found with cholesterol level and the dispensing of Simvastatin. Interestingly, a high focus on blood work is shown with 8 of the top 10 event codes shown being lab results of blood work.

**TABLE 6:** Logistic Regression Comparison Using Reduced Feature Selection Results

Metric	ECNN Features			Full Features			P-Value
	Mean±Std. Dev.	95% CI		Mean±Std. Dev.	95% CI		
TPR	0.685±0.008	0.679	0.691	0.286±0.035	0.260	0.313	4.02E-16
TNR	0.711±0.009	0.705	0.718	0.804±0.016	0.792	0.816	0.014
PPV	0.746±0.010	0.738	0.754	0.487±0.032	0.463	0.511	4.63E-14
NPV	0.646±0.007	0.640	0.651	0.633±0.024	0.615	0.651	1.84E-09
Accuracy	0.697±0.005	0.693	0.701	0.600±0.018	0.586	0.613	2.82E-15
AUROC	0.719±0.007	0.717	0.721	0.386±0.034	0.376	0.397	2.68E-61

Shown is a comparative analysis of two logistic regression classification models, one trained on ECNN feature selections and one on the full set of dataset features. As shown, ECNN feature selections result in superior classification performance whilst providing a significant reduction in feature size.

**TABLE 7:** ECNN and Random Forest Reduced Feature Selection Results

Metric	ECNN Features		RF Features				Random Selection Features	
	NN	RF	NN	P-Value	RF	P-Value	NN	RF
TPR	0.758±0.014	0.721±0.0008	0.775±0.039	0.077	0.720±0.003	0.125	0.998±0.002	0.688±0.540
TNR	0.759±0.025	0.780±0.0012	0.659±0.056	2.76E-11	0.743±0.002	2.00E-79	0.002±0.002	0.323±0.559
PPV	0.766±0.016	0.773±0.0008	0.704±0.024	0.06E-19	0.745±0.001	1.21E-90	0.510±0.001	0.566±0.098
NPV	0.751±0.005	0.728±0.0004	0.739±0.017	0.002	0.719±0.002	1.42E-36	0.308±0.314	0.233±0.251
FPR	0.241±0.025	0.220±0.0011	0.341±0.056	2.10E-12	0.257±0.002	1.50E-78	0.998±0.002	0.677±0.559
FNR	0.242±0.014	0.280±0.0008	0.225±0.039	0.062	0.280±0.003	0.490	0.002±0.002	0.312±0.540
Accuracy	0.759±0.006	0.750±0.0003	0.718±0.007	8.71E-36	0.732±0.001	1.70E-79	0.510±0.001	0.509±0.001
F1 score	0.762±0.002	0.746±0.0003	0.737±0.005	2.15E-38	0.732±0.001	3.82E-73	0.675±0.001	0.489±0.322
AUROC	0.854±0.015	0.785±0.0008	0.734±0.031	4.54E-30	0.735±0.002	6.32E-91	0.591±0.002	0.604±0.532

Shown is a comparison of feature selection effectiveness between ECNN, random forest - a similar traditional feature selection methodology, and random feature selection. Two types of basic classification models: neural network and random forest, trained on the various feature selection subsets, are used in determining selection effectiveness via predictive performance. A baseline random subset of features was also evaluated P-value analysis is a comparison of ECNN features against RF features of the corresponding underlying predictive model.

In a direct pair-wise comparison of predictive performance of feature ranking based on ECNN versus RF for each of the baseline NN and RF classification models shows generally superior performance using features ranked by ECNN. Highlighted by a 4.1% improvement in F1 score between RF and proposed when using a NN baseline classification model and a 1.4% improvement using a RF baseline classification model.

A definitive superior baseline model in an application standpoint for our feature subset use case however, is not as clear cut; with RF providing superior TNR with comparable accuracy scores and NN providing overall best F1 score and accuracy. In consideration of an application based hospitalization warning system, NN provides the superior NPV and as such, the superior screening type test for high risk demented patients.

In regards to the baseline random feature selection process, both feature selection methodologies produced results significantly improved over that of random guessing. Of note however, is the inability of NN in training an effective classification model when using the randomly selected feature subset, with final inactive models producing continuous positive predictions resulting in a ‘superior’ TPR. Additionally, RF also produced generally inactive models using the random feature subset, swinging between continuous positive or continuous negative predictions indicated by significantly large standard deviations. As such, random feature subset results do not provide an effective comparison of proportional predictive performance as compared to non-random feature selection methodologies but instead highlight the difficulties of selecting small subsets of features able to adequately model patient hospitalization.

In reference to table 6, feature ranking and selection using ECNN shows a statistically significant improvement in overall predictive performance as opposed to the use of the full feature dataset using a traditional logistic regression classification model. Said results highlight the challenges of such a high-dimensional and sparse dataset and the advantages of effective feature selection, enabling effective modelling of the problem space in a significantly reduced subset of features. Such complexity reduction is emphasised in the contrast of average training durations with logistic regression trained on the full set of features requiring 33 minutes whilst training on a subset of 10 features requiring seconds.

## VI. Conclusion

This study proposes a novel combination of methodologies for the prediction of hospitalisation potential with patients suffering from dementia. Using a novel adaption of snapshot ensembles to use a dynamically generated learning rate schedule, in addition to an adaption of entropy weight regularization for use with NNs and subsequent novel evaluation of model parameters: we were able to identify 10 medical events highly indicative of future hospitalization of demented individuals out of an extremely high dimensional and sparse dataset of 54,647 unique medical events. Comprising of diagnostic events, medication prescriptions and procedures, said events were able to model and predict future hospitalization to a performance equal (and in certain cases better) than that of the full dataset. ECNN provides significant advantages to statistical feature selection methods in interpretability and in ML based modelling techniques in predictive performance.

The identification of said medical events, opens avenues for the potential creation of early warning systems to identify demented individuals at high risk of hospitalization or institutionalization. With multiple indications of nutritional health being a major impact in hospitalization risk factor, such information can be further investigated for potential prevention through an emphasis in improved nutritional care for dementia patients. Such examples highlight the many possibilities focusing on pre-empting and preventing hospitalization through alteration of secondary care practices. Overall contributions such as those indicated allow for a potential reduction in critical healthcare utilization, itself a positive advancement, whilst reducing risk in a statistically elderly and vulnerable population through reduction in exposure to hospital induced risk factors such as infection.

Multiple avenues exist for the improvement of ECNN as future work. Most significantly would be the inclusion of times-series based modelling methods able to acknowledge the continually changing health of the individual patient over time. Further avenues of improvement also include greater statistical analysis of ranked features for improved ranking, larger scale datasets extending coverage beyond the Wales region currently handled by secure anonymised information linkage (SAIL), and adoption of state-of-the-art deep learning modelling such as event code representation using word embedding techniques for overall improved prediction performance. Additional application can be considered through the use of more novel modelling methodologies such as evolutionary algorithms to at once compare predictive performance whilst simultaneously providing feature reduction.

The collection of medical events highlight already established risk factors for hospitalization indicating effective capability whilst novel events present opportunity for further focused traditional clinical analysis as potential risk factors and indicators. As such, ECNN provides future potential for use within other medical informatics domains as risk factor identification. The general nature of patient medical records, in conjunction with ECNN enables application within other domains to provide interpretable, small-scale indicators allowing for ease of identification of at risk individuals for pre-emptive care.
